# Detection of Neolithic Settlements in Thessaly (Greece) Through Multispectral and Hyperspectral Satellite Imagery

**DOI:** 10.3390/s90201167

**Published:** 2009-02-23

**Authors:** Dimitrios Alexakis, Apostolos Sarris, Theodoros Astaras, Konstantinos Albanakis

**Affiliations:** 1 Remote Sensing and GIS Applications Laboratory, Department of Physical and Environmental Geography, School of Geology, Aristotle University of Thessaloniki, Thessaloniki 54124, Greece; 2 Laboratory of Geophysical – Satellite Remote Sensing & Archaeo-environment, Institute for Mediterranean Studies, Foundation for Research & Technology, Hellas (F.O.R.T.H.), Rethymno 74100, Crete, Greece

**Keywords:** Remote Sensing, Hyperspectral Imagery, Tells, Thessaly, Neolithic

## Abstract

Thessaly is a low relief region in Greece where hundreds of Neolithic settlements/tells called *magoules* were established from the Early Neolithic period until the Bronze Age (6,000 – 3,000 BC). Multi-sensor remote sensing was applied to the study area in order to evaluate its potential to detect Neolithic settlements. Hundreds of sites were geo-referenced through systematic GPS surveying throughout the region. Data from four primary sensors were used, namely Landsat ETM, ASTER, EO1 - HYPERION and IKONOS. A range of image processing techniques were originally applied to the hyperspectral imagery in order to detect the settlements and validate the results of GPS surveying. Although specific difficulties were encountered in the automatic classification of archaeological features composed by a similar parent material with the surrounding landscape, the results of the research suggested a different response of each sensor to the detection of the Neolithic settlements, according to their spectral and spatial resolution.

## 1. Introduction

The spectral capability of early satellite sensors opened new perspectives in the field of archaeological research. The recent availability of hyperspectral and multispectral satellite imageries has established a valid and low cost alternative to aerial imagery in the field of archaeological remote sensing. The high spatial resolution and spectral capability can make the VHR satellite images a valuable data source for archaeological investigation, ranging from synoptic views to small details [1].

Since the beginning of the 20th century, aerial photography has been used in archaeology primarily to view features on the earth's surface, which are difficult if not impossible to visualize from the ground level [2-4]. Archaeology is a recent application area of satellite remote sensing and features such as ancient settlements can be detected with remote sensing procedures, provided that the spatial resolution of the sensor is adequate enough to detect the features [5].

A number of different satellite sensors have been employed in a variety of archaeological applications to the mapping of subsurface remains and the management and protection of archaeological sites [6-11]. The advantage of satellite imagery over aerial photography is the greater spectral range, due to the capabilities of the various on-board sensors. Most satellite multi-spectral sensors have the ability to capture data within the visible and non-visible spectrum, encompassing a portion of the ultraviolet region, the visible, and the IR region, enabling a more comprehensive analysis [12]. Multispectral imagery such as Landsat or ASTER is considered to be a standard means for the classification of ground cover and soil types [13]. Concerning the detection of settlement mounds the above sensors have been proven to be helpful for the identification of un-vegetated and eroded sites [5]. In recent years the high spatial resolution imageries of IKONOS and Quickbird have been used for the detection of settlements and shallow depth monuments [14-16]. Hyperspectral imagery (both airborne and satellite) has been also applied in archaeological investigations on an experimental basis and need further investigation [2,17,18].

The goal of this particular project was the application of different methods and means of satellite remote sensing for the detection of Neolithic settlements. In this study four satellite remote sensing images with different spatial resolutions (ASTER, Landsat, HYPERION, IKONOS) were examined in order to search their potential for automatic extraction of Neolithic settlements, by means of pixel – based and object – based methods. This paper seeks to address these issues through a multi – sensor case study in Thessaly, Greece, where different satellite image processing techniques contributed to the detection of the so called ‘magoules’ that are found in the Thessalian plains. The satellite data were statistically analyzed, together with other environmental parameters, to examine any kind of correlation between environmental, archaeological and satellite data. Moreover, different methods were compared and integrated methodologies for the detection of Neolithic settlements were extracted. The results of the study suggested that the complementary use of different imagery can provide more satisfactory results.

## 2. Study Area and Data

**Figure 1.**
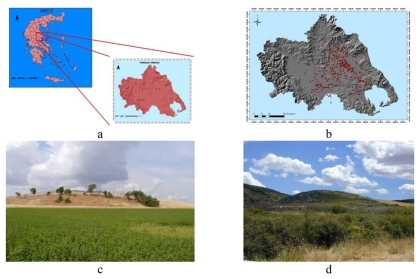
The region of Thessaly is located at the center of the mainland of Greece (Top Left). Most of the magoules are distributed within the limits of the plains of Larisa and Karditsa (Top Right). Details of the magoula of Kastro (Bottom Left). Details of the magoula of Kalo Nero (Bottom Right).

Thessaly is a relatively closed geographical unit, with definite mountainous borders (Mt. Antichasia and Olympus in the north, Mt. Ossa, Mavrovouni and Pelion in the east, Mt. Othris in the south, and Mt. Pindus in the west, reaching heights of 2,000 m) and two accesses to the sea, one through the Tempe gorge (NE) and another between the Othrys and Maurovouni mountains to the gulf of Volos.. All Thessalian basins show continuous habitation during all phases of the Neolithic period. As a matter of fact, Thessaly is famous for its long-lasting sites on its extensive fertile soils (Figure 1). The Neolithic settlement mounds are typically low hills of 1–5 meters height and a mean diameter of 300 meters, mainly consisting of loam and mud based materials. There are hundreds of Neolithic settlements/tells called magoules all over Thessaly, with different kind of vegetation now above them. Due to the intensive cultivation of the land in the past, not all of them are visible. Past field archaeological surveys were able to identify a number of them based mainly on the surface concentration of sherds and lithic material [19-22]. However most of the magoules (137) are mainly at East Thessaly (Larisa Plain) and less (63) in west Thessaly (Karditsa Plain). These two plains consist of Quaternary alluvial deposits.

The study involved satellite image detection of Neolithic Settlements in Thessaly by incorporating the following satellite and digital spatial data (Table 1):

- 4 ASTER images.
- 1 Landsat ETM image.
- 1 HYPERION image: Only 137 of the 242 total HYPERION bands were used in the analysis, because many of the bands exhibited low signal to noise ratio or other problems.
- 4 IKONOS images: For each image, the multispectral bands were fused with the high resolution panchromatic band in order to exploit the spectral information of the four multispectral bands (blue, green, red, near infrared) and the effective spatial resolution of the panchromatic band.
- 18 Air photos acquired from the Geographic Service of the Hellenic Army – GYS.
- The results of topographic mapping through systematic GPS surveying of more than 342 Neolithic settlements of Thessaly.
- A DEM of 20 m pixel size of the study area. The DEM was constructed after digitizing in GIS environment 24 topographic maps scale 1:50.000 from the Geographic Service of the Hellenic Army. It has to be mentioned that ASTER DEM was also exploited in the particular study but it did not cover sufficiently the whole area of interest, and second, the specific images have different area coverage and only the ASTER mosaic was able to cover the whole region of Thessaly.

## 3. Research Methodology and Results

The image processing of satellite data was carried out in two steps starting with the basic preprocessing procedures followed by more sophisticated image processing steps.

### 3.1. Preprocessing of Satellite Images

**Figure 2.**
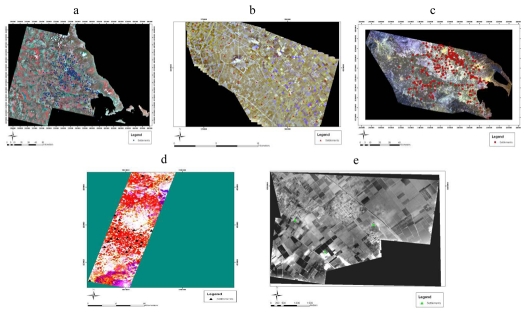
(a) Mosaic of ASTER images; (b) Mosaic of IKONOS images; (c) Landsat Image; (d) HYPERION image; (e) Mosaic of airphotos.

The construction of image mosaics (Figure 2) followed the masking of the sea, the clouds and the snow areas using Erdas Imagine 9.1 software package.

The next step had to do with the transformation of the projection systems of all images to the Hellenic Geodetic System of Reference (EGSA87/HGSR87) so that they can all be fused to the same projection system. The final step of image preprocessing was the conversion of DN (Digital Number) values of images to reflectance. Different equations to convert the DN values to radiance were employed. The conversion of the DN values of ASTER images was achieved through the equation: Lrad = (DN-1)*Unit Conversion Coefficient [23]. For the IKONOS images the equation: *Lrad = DN/Unit Conversion Coefficient* was used [24]. The conversion of DN values of Landsat images to radiance was accomplished through the equation: Lrad = DN * Grescale + Brescale where *Grescale and Brescale* are band specific rescaling factors [25]. For the case of HYPERION images “signal to noise” ratio was used to select 137 bands from the total of 242. Then DN values were converted to radiance values according to the equations: *VNIRL = DN /40, SWIRL = DN / 80* (USGS, 2007). The last conversion had to do with the conversion of the radiance of all images to reflectance through the general algorithm by [26] (1):

(1) $${\text{P}}_{\text{p}}=\Pi {\text{L}}_{\lambda }{\text{d}}^{2}/\text{ESUN}\lambda \text{cos}\theta_{\text{s}}$$

where :

P_p_ unitless planetary reflectance
L_λ_ spectral radiance at the sensor's aperture
d^2^ earth–sun distance in astronomical units
ESUN_λ_ mean solar exoatmospheric irradiances
Θ_s_solar zenith angle in degrees.

### 3.2. Composition of RGB Composites

**Figure 3.**
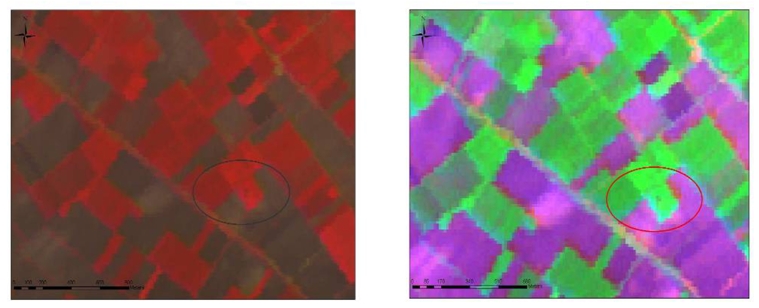
RGB→3,2,5 of ASTER image –Melisa Settlement 1 (left). RGB→2,3,7 of ASTER image – Melisa Settlement 1 (right).

Several RGB composites were constructed in an effort to examine their efficiency in the detection of the Neolithic settlements. For the ASTER image with acquisition date 19-03-2003, where most of the magoules are registered, the RGB→1,2,3, RGB→3,2,5 and RGB→2,3,7 composites (Figure 3) were the most successful for the visual detection of the Neolithic settlements (Out of 239 settlements, 39 of them were highly visible, 49 average visible and 151 poorly visible). Those composites appeared to have the highest Optimum Index Factor. High OIF values indicate bands that contain much “information” with little correlation. By using the OIF method, three band components of an RGB can be evaluated on their effectiveness for display [27]. OIF is defined by equation (2).

(2) $$\text{OIF}=\text{Max}\left[\sum_{\text{i}=1}^{\text{n}}\text{s}(\text{i})/\sum_{\text{i}=1}^{\text{n}}\left|\text{r}(\text{ij})\right|\right]$$

where s _i_ is the standard deviation of band i and r(ij) is the correlation coefficient of band i and band j.

**Figure 4.**
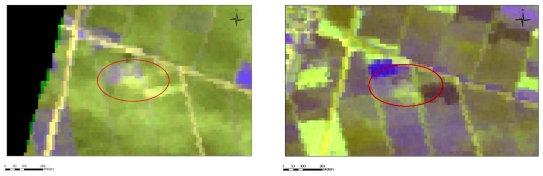
Appearance of the Orfana settlement on the ASTER image (RGB→1,2,3) with acquisition date of 19-03-2003 (left). Right: Appearance of the same settlement on 30- 06- 2004 (right).

Similarly, RGB composites of IKONOS images were able to detect 27 in a total of 48 settlements. It has to be noted that 19 of the detectable magoules, namely the highest of all corresponding to an average altitude of 4.6 m, were highly visible in all RGB composites. On the other hand, RGB composites of Landsat and HYPERION images were not very promising (for HYPERION composites only five settlements were detected in a total of 21). Finally, average altitude aerial images contributed to an excellent detection of all the five settlements that were inside the spatial limits of the airphoto mosaic. As a general conclusion however, the most crucial factors for the detection of magoules proved to be the acquisition date of the image due to the fact that the land around the majority of the settlements is cultivated (Figure 4).

**Figure 5.**
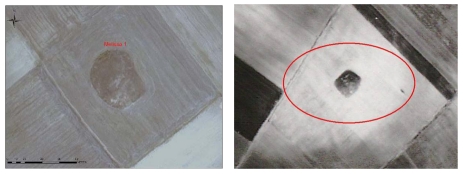
IKONOS image. RGB – 321 - Melissa 1 Settlement (left). Melia 2 Settlement –Airphoto image (right).

Visual interpretation is commonly used for visual extraction of obvious and large or medium scale archaeological structures like settlement mounds [28-30]. For IKONOS images it was possible to detect most of the settlements with just a simple visual interpretation of any kind of RGB composite due to the high spatial resolutions of the specific image. The visual detection of them was achieved based on shape, linearity, tone, and texture size between different patterns around them [14]. The same task was accomplished for the airphotos. However, the lack of airphoto data and their small spatial coverage of the study area turned air photos to have ancillary role in the whole study (Figure 5).

### 3.3. Spectral Profile Comparison and Classification

**Figure 6.**
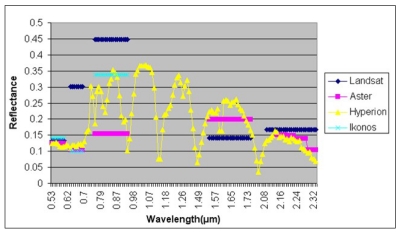
Comparison of spectral signatures of all the sensors from the Neolithic settlements collected from the plains of Thessaly.

The identification of spectral signatures was considered to be a crucial task for the detection of Neolithic settlements especially for the classification process. That task was accomplished in order to exploit any potential distinct spectral characteristics of surface and subsurface settlements patterns compared with the surrounding material [2]. Signatures were collected from all tells and were divided into two categories: those collected from plain areas and those collected from mountainous areas due to different soil cover (Figure 6).

**Figure 7.**
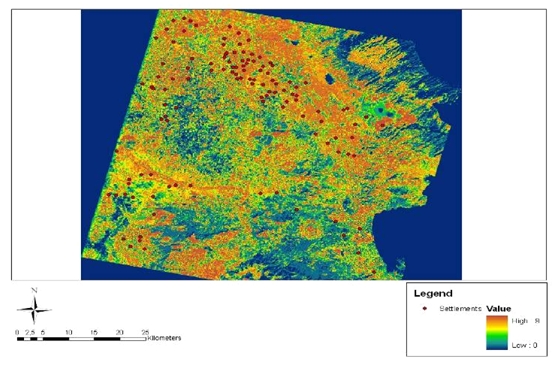
Classification map from the spectral signatures of ASTER images.

The basic statistics for each band for all satellite images have been evaluated. Each band was reclassified in two categories: a) for all pixels within the range of <reflectance>+/-σ and b) for all the pixels outside the specific range. As a result, binary files were created and Boolean addition in GIS environment was followed to produce a final classification map (Figure 7).

After the creation of the spectral signature modeling map, 64 settlements in a total of 120 (56.6%) were established in areas of very high possibility.

### 3.4. Principal Component Analysis

**Figure 8.**
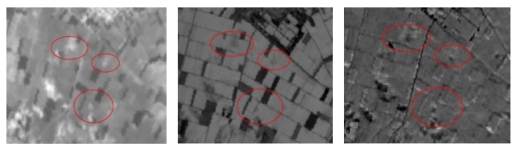
Appearance of three settlements to the first Principal Component of ASTER image (left). Appearance of three settlements to the second Principal Component of ASTER image (middle). Bottom Appearance of three settlements to the third Principal Component of ASTER image (right).

Principal Component Analysis involves a mathematical procedure that transforms a number of correlated variables in a smaller number of uncorrelated variables called principal components. The method was applied to ASTER, Landsat and HYPERION images to decorrelate the data and to reduce the dimension of the study [31]. PCA of ASTER images concluded to the best results where 39 settlements were highly discriminated and 47 medium discriminated in a total of 247. Furthermore, 14 magoules that were not visible in the original images were clearly visible after applying PCA to ASTER images (Figure 8).

### 3.5. Data Fusion

**Figure 9.**
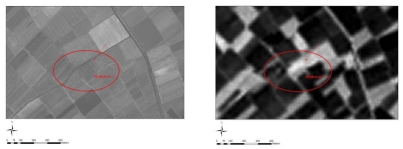
Settlement Moshohori represented in an IKONOS image (left) and the same region after image fusion between IKONOS and HYPERION (right).

Image fusion is a standard satellite image procedure of combining images of different spatial resolution to obtain a single final composite image. Image fusion is applied to digital imagery for different reasons such as to enhance certain features that are not visible in either of the single data alone [1] and to sharpen the images [32]. The images that can be used can be from different sensors and resolutions. By using ERDAS imagine software various fusion combinations and techniques were tried, such as ASTER (15 m) visible channels with the PCA product (PC1) of HYPERION (30 m) or the high resolution (1 m) bands (datafusion products) of IKONOS with the PCA product (PC1) of the HYPERION. PC1 of HYPERION image was selected in order to exploit the best radiometric resolution available compared to the rest high spatial resolution images. The results were highly promising for the cases of fusion (using PCA technique, namely re-scaling the high resolution image to fit the data range of PC1 following an inverse PC transformation, and cubic convolution interpolation) between high spatial resolution and high spectral resolution images (Figure 9).

### 3.6. Spectral Mixer Utility

**Figure 10.**
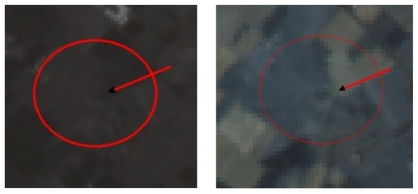
Appearance of a settlement RGB→ 8, 9, 10 (left) Appearance of the same settlement after application of RGB1 (right).

In our effort to exploit the high spectral resolution of HYPERION images, a spectral mixer application through the use of Erdas Imagine 9.1 software was also applied. Spectral Mixer produces three bands to be assigned to the red, green, and blue color guns, but in this case instead of just assigning each band to a color gun one can select a weighted average of spectral bands to be assigned to a color gun [33]. For HYPERION images only the bands that had reflectance values above 0.3 were chosen and a weighting coefficient of 0.14 was applied for each band. The new RGB that was created (RGB1) employed the mixing of the bands (38, 42, 48, 49, 50, 51, 52), (85, 86, 87, 88, 89, 90, 91, 92,) and (93, 94, 108, 109, 110, 111, 113, 114) (Figure 10).

### 3.7. Radiometric Enhancement

**Figure 11.**
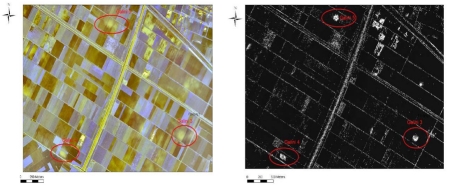
Appearance of three settlements in the original IKONOS image (left) and the radiometrically enhanced image where three Neolithic settlements are highlighted (right). To the north of Galini-3 settlement, shown at the lower right of the image, another smaller potential magoula is suggested.

Due to the variable quality of the original images, the radiometric enhancement was vital for the appearance of the images and the better recognition of the terrain features. After applying radiometric enhancement to ASTER images (acquisition date of 19-03-2003) 57 settlements were detected. A non-linear radiometric enhancement of the HYPERION PCA image, followed by an inversion of brightness was able to highlight eight settlements from a total of nine. (Melia 1, Melia 2, Anagennisi 2, Moshohori 3, Kipseli 2, Prodromos 1 of Larisa, Nikaia 17 and Kuparissia 2). Similar type of non-linear radiometric enhancement of the high resolution IKONOS images through the modification of the histogram was able to outline the round shape of known magoules, as well as to identify 10 more targets of similar geometry that need to be verified by the ground truthing activities that will follow (Figure 11).

**Figure 12.**
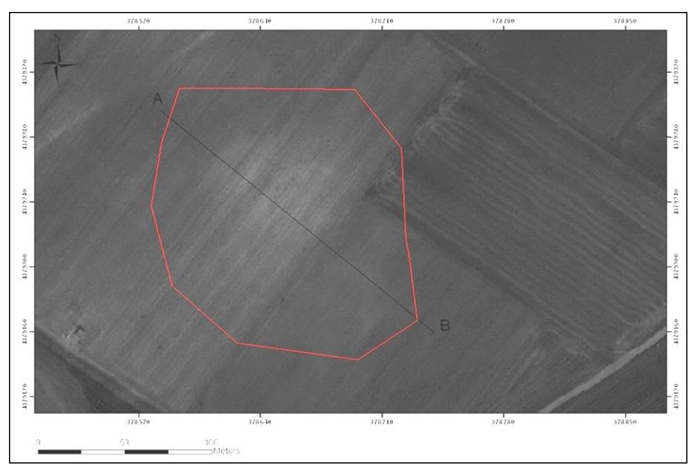
Appearance of the Anagennisi 2 Settlement to band 1 of IKONOS image.

The fact that different kinds of marks, such as crop, soil and shadow marks, are generally associated with the presence of buried archaeological remains [34-36] was exploited at the IKONOS images so as to detect some completely flat magoules such as Anagennisi 2 (Figure 12). Soil and moisture differences within near-surface archaeological deposits can influence surface vegetation patterns creating crop marks of various kinds. In addition soil marks can appear as changes in color or texture in freshly ploughed fields before the growing crops mask the surface of the soil [37].

### 3.8. Land Classification and Vegetation Indices

**Figure 13.**
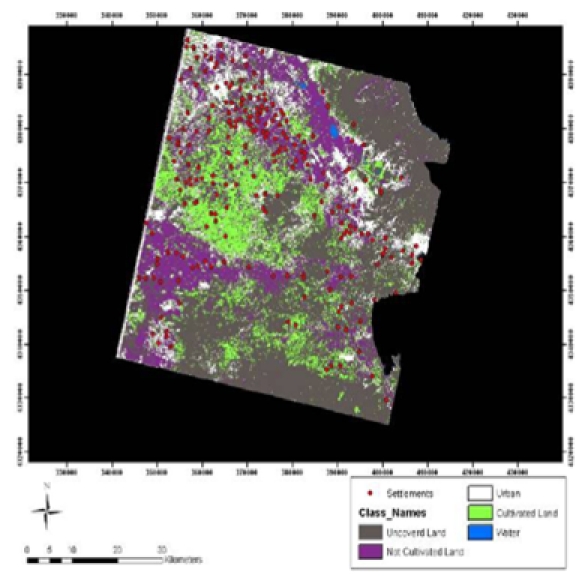
Results of the Land Classification of ASTER image through the use of Mahalanobis Distance (fuzzy) algorithm.

In most cases, difficulties in the detection of archaeological sites originate due to the fact that the spectral response of archaeological sites and surrounding areas is almost the same [2,14,38]. However, in the domain of predictive modeling, the specification of the environmental attributes that correlate to the location of the archaeological sites is of importance. For this reason, in order to investigate the regime of the land use surrounding the magoules, several methods of supervised classification were applied to Landsat and ASTER images. For the classification procedure five classes were defined: Uncovered land, Uncultivated land, Cultivated land, Urban area and Water reservoirs. Mahalanobis fuzzy classification proved to be the most efficient one in terms of the overall accuracy assessment (based on the error matrix) compared to all the classification algorithms that were applied (Maximum Likelihood, Minimum Distance, Mahalanobis Distance, Parallelepiped, Spectral Angle Mapper, Maximum Likelihood (fuzzy), Minimum Distance (fuzzy), Mahalanobis Distance (fuzzy)) (Table 2 and Figure 13).

Due to the small agreement between the land use classification results that produced between Landsat and ASTER sensors, the Normalised Difference Vegetation Index (NDVI) was computed to analyse the difference of vegetation during various acquisition dates. Vegetation indices are mainly extracted from reflectance data from the red and near infrared (NIR) bands [39]. The NDVI was obtained by the following equation (3):

(3) NDVI=[NIR−Red]/[NIR+Red]

**Figure 14.**
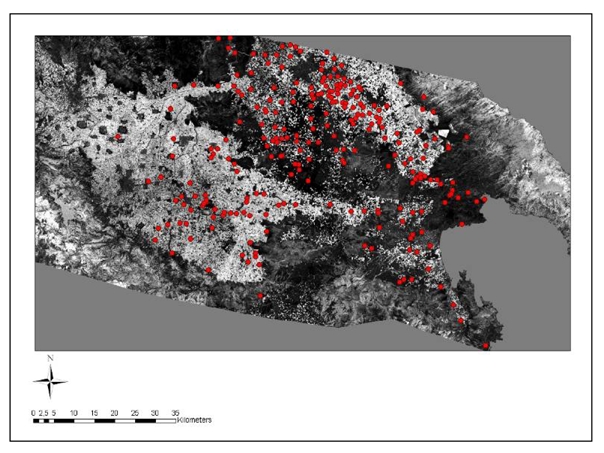
Application of NDVI to Landsat image. The Neolithic settlements appear as red dots.

As expected, the NDVI of the spring ASTER image was higher than the summer Landsat image (Figure 14).

### 3.9. De-correlation Stretch

**Figure 15.**
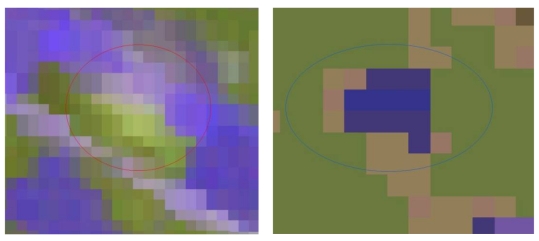
Original ASTER (RGB→1,2,3) image indicating the Galini 4 settlements (left) and the same area after the application of De-correlation Stretch (right).

The de-correlation stretch is a process that is used to enhance (stretch) the color differences found in the input pixels. The principal component transformation is similar, except the fact that the transformation vectors are derived from the correlation matrix rather than the covariance matrix. De-correlation stretch to the ASTER images managed not only to detect easily 36 Neolithic settlements (Figure 15), but also to estimate the area of each settlement in GIS environment.

### 3.10. Spatial Enhancement

**Figure 16.**
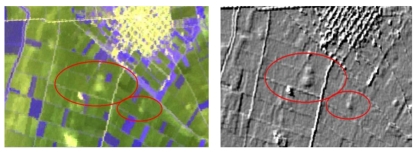

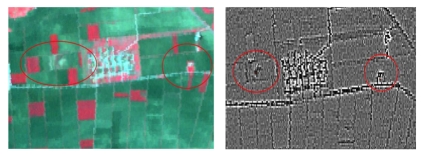
Original ASTER image (RGB→1,2,3) around Halki area (Top Left) and the corresponding image after the application of Sobel Right Diagonal filter (Top Right). Original ASTER image (RGB→1,2,3) around the settlements of Elliniko 1 and Elliniko 2 (Bottom Left) and the corresponding image after the application of Laplace Filter (Bottom Right).

Spatial enhancement of images is considered to be a standard satellite image enhancement. In order to emphasize the marks arising from the presence of magoules to Thessaly plain various spatial filters were applied to all the images. Of the several types of filters that were applied in the specific study, only two of them, Sobel Right Diagonal 3×3 and Laplace 3×3, proved to be very useful for the detection of Neolithic settlements. Although the spatial filters were applied to all bands of ASTER and the first three principal components of HYPERION images, they were especially satisfactory when they were applied at the first band of ASTER image (Figure 16).

The values of the matrices can be seen in Table 3. The extraction of statistics about the number of settlements that were detected by each filter indicated that Sobel right diagonal filter was the most reliable one achieving a discrimination of almost 150 sites (Table 4).

### 3.11. Object Based Remote Sensing

**Figure 17.**
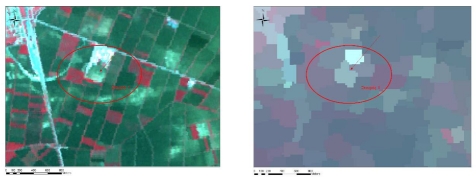
RGB – 3,2,1 ASTER image – Stauros 1 Settlement (left). Stauros 1 after the application of object oriented methodology (right).

The ASTER image was segmented and classified based on an object based approach through the use of e-Cognition software. The object based technique is considered as very useful for heterogeneous land covers [14]. Segmentation is the most important phase in object based classification. The image is subdivided to homogeneous areas based on their spatial characteristics, shape, scale and object hierarchy level [40]. The second phase includes the classification of image, where training objects are selected to train the classification in a similar way to the pixel based classification but instead of using pixels as training samples, geometric objects are used. Subsequently, classification parameters are defined [14]. The application of object – oriented methodology to the ASTER images managed to detect easily only 15 settlements in a total of 234, whereas 185 settlements were not discriminated at all and 34 were medium discriminated from the neighbor pattern. For the application of segmentation to ASTER image we used a scale factor of 5. However, the fact that the settlements don't have uniform shape and spatial characteristics was the main reason for the poor results of this methodology (Figure 17).

## 4. Predictive Modeling

**Figure 18.**
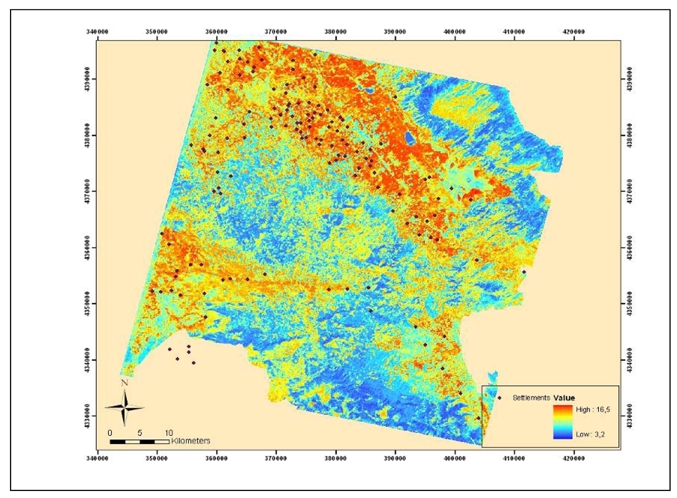
Map of predictive modeling.

After applying all the above enhancement processes a predictive model was designed to locate potential magoules in the wider region of the Thessaly plain. The results of land use classification, NDVI estimates and those from the spectral signatures and classification of the ASTER image (acquisition date 19-03-2003) were combined together with a DEM constructed by digitization of 1:50.000 scale topographic maps. All these data were reclassified and a certain weight factor was applied to each cell of the raster layers. The weighting and rating factors were specified based on the statistical analysis of the specific parameters in relation to the correlation of them with the known magoules and their importance in terms of the location of the magoules. All the raster layers were rated (Table 5) and equation (4) was used through the raster calculator of ArcGIS 9.1 software to construct the final predictive model map (Figure 18):

(4) Predictive Areas=DEM∗0.3+Land Use∗0.5+NDVI∗0.3+Spectral Signature Map∗0.7

The final predictive map consisted of pixel areas with different probability for the existence of Neolithic settlements. It was estimated that 92 of the already known settlements are laid on areas of high probability and 23 in areas of medium probability.

## 5. Conclusions

The various approaches applied on different satellite images for the detection of Neolithic settlements in Thessaly illustrate the benefits that satellite remote sensing can provide in archaeological investigations. It was proven that an integration of images from different satellite sensors can contribute to a faster and more accurate and qualitative detection of archaeological sites.

Specifically, ASTER images proved to be the most reliable and efficient for the detection of Neolithic settlements, being able to combine a medium spatial resolution with high spectral resolution. In contrast, Landsat images concluded to quite poor results, mainly due to the acquisition date of the imagery, which produced low signal to noise ratio for the archaeological targets. The high spectral abilities of HYPERION especially after merging it with the high resolution images of IKONOS seem to have an increased potential not only in detecting but also in outlining the particular features. The image processes that proved to be more effective were the spatial filtering, the process of de-correlation stretch and the radiometric enhancement. The integration of land use classification data with NDVI and spectral signatures resulted to very promising modeling maps. On the other hand, the object based classification method proved that most of Neolithic mounds lack uniform shape characteristics that can be easily distinguished from the surrounding vegetation patterns. Although most of them have a circular or oval shape, they belong to the same land use type of the wider region that makes them almost impossible to separate from the other features of the terrain. Furthermore, although the use of conventional aerial photos can often pinpoint particular features based on crop marks, the intensive use of space and the relatively leveling of the ground at the magoules has masked their particular features. This makes them impossible to be identified without the exploitation of the enhanced radiometric resolution of satellite imagery. Thus, satellite remote sensing may offer further advantages with other type of archaeological targets, and it offers potential for further investigation. The vegetational regime at the mounds proved to be a crucial factor for their detection. In case there had been different kinds of vegetation on the settlements and the surrounding areas, the automatic extraction by means of remote sensing would have been easier.

The above processes were limited to the satellite imagery. The particular methods can be also employed for the detection and mapping of similar archaeological targets such as Bronze Age mounds and settlements, monumental tholos tombs and others. The results of this study can be further enhanced through manipulation of the above conclusions with the spatial tools of GIS applied to the distribution of the magoules on the geomorphologic attributes of the terrain. In this way, a more integrated and synthetic tool for the detection of the magoules and the study of the Neolithic settlement patterns can be produced.

## Figures and Tables

**Table 1.** Spectral, spatial and temporal attributes of the satellite sensors and air photos that were used for the study.

**Sensor**	**Acquisition Date**	**Spatial Resolution (m)**	**Subsystem**	**Band range (nm)**	**Band widths (nm)**	**Number of Spectral Bands**	**Radiometric Resolution**
**Hyper- Spectal Sensor**							
**1. HYPERION**	September 3, 2001	30	VNIR, SWIR	VNIR : 9-57 SWIR: 82-97, 101-119 135-164, 191-218	10 nm wide (approx) for all 196	137	16-bit
**Hyper - Spatial Sensors**							
**1. IKONOS**	June 1, 2006 December 12, 2005 March 1, 2007 December 12, 2005 June 16, 2006	1	VNIR	445 -516	71	4	11-bit
				506-595	89		
				632-698	66		
			SWIR	757-853	96		
**Multi - Spectral Sensors**							
**1. Landsat - 7 ETM+**	July, 28, 1999	30	VNIR	450-515	65	8	8 -bit
				525-605	80		
				630-690	60		
			SWIR	750-900	150		
				1550-1750	200		
				2090-2350	260		
		60	TIR	1040-1250	210		
		15	Panchromatic	500-900	400		
**2. ASTER**	March, 19, 2003 June, 30, 2004 June, 30, 2003 March, 19, 2003	15	VNIR	520-600	80	14	8-bit
				630-690	60		
				780-860	80		
		30	SWIR	1600-1700	100		
				2145-2185	40		
				2185-2225	40		
				2235-2285	50		
				2295-2365	70		
				2360-2430	70		
		90	TIR	8125-8475	350		12-bit
				8475-8825	350		
				8925-9275	350		
				10250-10950	700		
**Air photos**	January 3, 1999, 18 air photos						

**Table 2.** Accuracy of each image classification method.

**Classification Method**	**Overall Accuracy (%)**

Minimum Distance	78
Mahalanobis	80
Maximum Likelihood	84
Maximum Likelihood (fuzzy classification)	90
Mahalanobis (fuzzy classification)	96
Minimum Distance (fuzzy classification)	89
Spectral Angle Mapper	59
Parallilepiped	90

**Table 3.** Sobel Right Diagonal 3×3 (left). Laplace Filter (right).

*-2*	*-1*	*0*	1	4	1
*-1*	*0*	*1*	4	-20	4
*0*	*1*	*2*	1	4	1

**Table 4.** Statistical analysis of the number of settlements that were enhanced after the application of different filters to various images. The grade of discrimination depended on the visual recognition and detection of the magoules.

	Sobel Filter		Laplace Filter	
**ASTER (Larisa Area)**	Number of Settlements	Height (mean –meters)	Number of Settlements	Height (mean –meters
Excellent Discrimination	59	4.37	40	5.15
Medium Discrimination	86	3.92	48	4.12
Bad Discrimination	69	3.51	121	3.14
Sum	211		211	
	Sobel Filter		Laplace Filter	
**HYPERION (PCA 1)**	Number of Settlements	Height (mean –meters)	Number of Settlements	Height (mean –meters
Excellent Discrimination	6	3.8	-	-
Medium Discrimination	6	4.33	-	-
Bad Discrimination	7	3.57	-	-
Sum	19		-	-
	Sobel Filter		Laplace Filter	
**ASTER (Karditsa Area**	Number of Settlements	Height (mean –meters)	Number of Settlements	Height (mean –meters
Excellent Discrimination	3	2.33	0	0
Medium Discrimination	12	3.66	7	4.57
Bad Discrimination	31	4.9	39	4.41
Sum	46	4.43	46	4.43

**Table 5.** Weights and rating for each factor.

**FACTORS**	**WEIGHTING**	**RATING**

**DEM**		

Height < 120 m	9	0.3
120 – 200 m	6	
> 200 m	4	

**NDVI**		

> 0.2	8	0.5
0.2 – 0.3	6	
< 0.3	4	

**LAND USE**		

Uncovered Land	7	0.5
Urban	6	
Cultivated Land	5	
Not Cultivates Land	4	

**SPECTRAL SIGNATURES**	From 1-9	0.7

## References

[1] Leckie D.G. (1990). Synergism of SAR and visible/infrared data for forest type discrimination. Photogramm. Eng. Remote Sens..

[2] Rowlands A., Sarris A. (2006). Detection of exposed and subsurface archaeological remains using multi – sensor remote sensing. J. Archaeol. Sci..

[3] McGovern P.E., Sever T.L., Myers J.W., Myers E.E., Bevan B., Miller N.F., Bottema S., Hongo H., Meadow R.H., Kuniholm P.I., Bowman S.G.E, Leese M.N., Hedges R.E.M., Matson F.R., Freestone I.C., Vaughan S.J., Henderson J., Vandiver P.B., Tumosa C.S., Beck C.W., Smith P., Child A.M., Pollard A.M., Thuesen I., Sease C. (1995). Science in Archaeology: A Review. Am. J. Archaeol..

[4] Vermeulen F., Verhoeven G. (2004). The contribution of aerial photography and field survey to the study of urbanization in the Potenza valley (Picenum). J. Roman Archaeol..

[5] Menze B.H., Sherratt A.G. (2006). Detection of Ancient Settlement Mounds: Archaeological Survey Based on the SRTM Terrain Model. Photogramm. Eng. Remote Sens..

[6] Cooper F.A., Bauer M., Cullen B., Behrens C., Sever T. (1991). Satellite spectral data and archaeological reconnaissance in western Greece. Applications of Space Age Technology in Anthropology.

[7] Custer J.F., Eveleigh T., Klemas V., Wells I. (1986). Application of LANDSAT data and synoptic remote sensing to predictive models for prehistoric archaeological sites: An example from the Delaware coastal plain. Amer. Ant..

[8] Ebert J.I., Lyons T.R. (1980). Remote sensing in archaeology, cultural resources treatment and anthropology: The United States of America in 1979. Aerial Arch..

[9] Liu J., Xu L., Sarris A., Topouzi S., Doerr M., Sarris A. (2003). CRM and archaeological research using remote sensing and GIS: Zhouyuan (China) and Lasithi (Greece).

[10] Madry S.L.H., Crumley C.L., Allen K.M.S., Green S.W., Zubrow E.B.W. (1990). An application of remote sensing and GIS in a regional archaeological settlement pattern analysis: the Arroux River valley, Burgundy, France. Interpreting Space: GIS and Archaeology.

[11] Sarris A., Weymouth J., Cullen B., Stein C., Wiseman J.T. (1996). The Nikopolis Project. Integration of geophysical prospection, satellite remote sensing, and GIS techniques in the study of Epirus, Greece.

[12] Pavlidis L. High resolution satellite imagery for archaeological application. www.fungis.org/images/newsletter/2005-1.pdf.

[13] Fowler M.J.F. (2002). Satellite Remote Sensing and Archaeology: a Comparative Study of Satellite Imagery of the Environs of Figsbury Ring, Wiltshire. Archaeol.Prospec..

[14] De Laet V., Paulissen E., Waelkens M. (2007). Methods for the extraction of archaeological features from very high-resolution IKONOS-2 remote sensing imagery, Hisar (southwest Turkey). J. Archeol. Sci..

[15] Masini N., Lasaponara R. (2007). Investigating the spectral capability of Quickbird data to detect archaeological remains buried under vegetated and not vegetated areas. J. Cult. Herit..

[16] Sarris A. (2005). Use of remote sensing for archaeology: state of the art.

[17] Cavalli R.M., Colosi F., Palombo A., Pignatti S., Poscolieri M. (2007). Remote Hyperspectral Imagery as a support to Archaeological Prospection. J. Cult. Herit..

[18] Merola P., Allegrini A., Guglierra D., Sampieri S. (2006). Buried Archaeological Structures Detection Using MIVIS Hyperspectral Airborne Data.

[19] French D.H. (1968). Anatolia and the Aegean in the Third Millenium B.C.. Ph.D. dissertation.

[20] Gallis K. (1992). Atlas proistorikon oikosmon tis anatolikis Thessalikis pediadas.

[21] Halstead P.L.J. (1984). Strategies for Survival: An Ecological Approach to Social and Economic Change in the Early Farming Communities of Thessaly, Northern Greece. Ph.D. dissertation.

[22] Wace A.J.B., Thompson M.S. (1912). Prehistoric Thessaly.

[23] Smith A. How to convert ASTER Radiance value to reflectance.

[24] Fleming D. (2001). IKONOS DN Value Conversion to Planetary Reflectance Values.

[25] Chander G., Marcham B. (2003). Revised Landsat – 5 TM Radiometric Calibration Procedures and Postcalibration Dynamic Ranges. IEEE Trans. Geosci. Remot. Sen..

[26] USGS How are the radiance values (L) determined within the HYPERION bands?. http://eo1.usgs.gov/faq.php?id=23.

[27] Buhe A., Tsuchiya K., Kaneko M., Ohtaisi N., Mahmut H. (2007). Land Cover of Oases and forest in XinJiang, China retrieved from ASTER data. Adv. Space Res..

[28] Altaweel M. (2005). The use of ASTER satellite imagery in archaeological contexts. Archaeol. Prospect..

[29] Parcak S. (2007). Satellite remote sensing methods for monitoring archaeological tells in the Middle East. J. Field Archaeol..

[30] Ur J. (2003). Corona satellite photography and ancient road networks: a northern Mesopotamian case study. Antiquity.

[31] Richards J.A., Xiuping J. (1998). Remote Sensing Digital Image Analysis.

[32] Chavez P.S., Sides S.C., Anderson J.A. (1991). Comparison of three different methods to merge multiresolution and multispectral data: TM & SPOT pan. Photogramm. Eng. Remote Sens..

[33] Erdas Inc. (2006). Erdas Field Guide..

[34] Bradford J. (1949). Buried landscapes in Southern Italy. Antiquity.

[35] Beck A. Archaeological site detection: the importance of contrast.

[36] Bewley R.H. (2003). Aerial survey for archaeology. Photogramm. Rec..

[37] Lasaponara R., Masini N. (2006). On the potential of Quickbird data for archaeological prospection. Int. J. Remote Sens..

[38] Boehler W., Heinz G., Qiming G., Shenping Y. (2004). The progress in satellite imaging and its application to archaeological documentation during the last decade.

[39] Lasaponara R., Masini N. (2007). Detection of Archaeological crop marks by using satellite Quickbird multispectral imagery. J. Archaeol. Sci..

[40] Giada S., De Groeve T., Ehrlich D. (2003). Information extraction from very high-resolution satellite imagery over Lukole refugee camp, Tanzania. Int. J. Remote Sens..

